# OR13-001 Loss-of-function mutations in CECR1, encoding adenosine deaminase 2 (ADA2), cause recurrent fevers and early onset strokes

**DOI:** 10.1186/1546-0096-11-S1-A263

**Published:** 2013-11-08

**Authors:** Q Zhou, A Zavialov, J Chae, M Hershfield, R Sood, S Burgess, A Zavialov, D Chin, M Gadina, R Goldbach-Mansky, A Ombrello, D Stone, I Aksentijevich, D Kastner

**Affiliations:** 1 Inflammatory Disease Section, NHGRI, Bethesda, USA; 2 Turku Centre for Biotechnology, University of Turku, Turku, Finland; 3 Medicine, Duke University Medical Center, Durham, USA; 4 Zebrafish Core, USA; 5 Developmental Genomics Section, NHGRI, USA; 6 Office of Science and Technology, NIAMS, Bethesda, USA; 7 Translational Autoinflammatory Disease Section, NIAMS, Bethesda, USA

## Introduction

Here we describe a new genetic disease characterized by fevers and systemic inflammation, livedo reticularis, mild immunodeficiency, and early-onset recurrent lacunar strokes in 5 unrelated patients.

## Objectives

To identify a possible genetic cause of this syndrome.

## Methods

We used a combination of exome and Sanger sequencing, SNP arrays, Western blots (WB), ELISA assays, radiochemical-thin layer chromatography, and HPLC.

## Results

The 5 unrelated patients shared 3 missense mutations in *CECR1*, encoding adenosine deaminase 2 (ADA2), with the genotypes A109D/Y453C, Y453C/G47A, G47A/H112Q, R169Q/Y453C, and R169Q/28kb genomic deletion encompassing the 5’UTR and first exon of *CECR1*. All of the mutations are either novel or present at low frequency (<0.001) in several large databases. Computer modeling based on the crystal structure of the human ADA2 protein suggests that *CECR1* mutations either disrupt protein stability or impair ADA2 enzyme activity. Whereas the ADA1 protein, which is mutated in some patients with severe combined immunodeficiency disease, is an intracellular protein that catalyzes the conversion of adenosine to inosine, ADA2 is expressed predominantly in myeloid cells and secreted into the blood, and has a lower affinity for adenosine than ADA1. Western blots showed a decrease in ADA2 protein in supernatants of PBMCs from patients relative to healthy controls. All patients had at least 10-fold diminished serum and plasma concentrations of ADA2 protein, and reduced ADA2-specific adenosine deaminase activity. However, whereas ADA1 deficiency leads to the accumulation of deoxyadenosine nucleotides and lymphocytotoxicity, such toxic metabolites were not found in the blood of patients with ADA2 deficiency. Animal models suggest that ADA2 is the prototype for a family of proteins with growth factor activity (adenosine deaminase growth factors, ADGF). There is no mouse homolog of *CECR1*, but there are 2 homologs in the zebrafish, *Cecr1a* and *Cecr1b*. While a zebrafish line with a hypomorphic retroviral insertion in *Cecr1a* did not exhibit an obvious phenotype, when we used 2 different morpholinos to knock down the expression of *Cecr1b*, at 48 hours post fertilization we observed intracranial hemorrhage in approximately 50% of zebrafish embryos, but in only 3% of control embryos. Utilizing zebrafish lines with GFP-tagged leukocytes, we found that *Cecr1b* knockdown also led to the near absence of neutrophils, but not monocytes. These observations suggest that the ADA2 homolog may be necessary both for vascular integrity and leukocyte development in the zebrafish, and that the near absence of ADA2 in humans may lead to strokes and autoinflammation by similar mechanisms.

## Conclusion

We propose the term *fever with early onset stroke* (*FEOS*) to denote this condition. Although it is a rare disease of children, FEOS may provide important insights into the role of ADGFs in human disease, and may elucidate novel pathways underlying strokes in adults.

## Disclosure of interest

None declared

